# Optimization of routine *KRAS* mutation PCR-based testing procedure for rational individualized first-line-targeted therapy selection in metastatic colorectal cancer

**DOI:** 10.1002/cam4.47

**Published:** 2013-02-03

**Authors:** Anne-Sophie Chretien, Alexandre Harlé, Magali Meyer-Lefebvre, Marie Rouyer, Marie Husson, Carole Ramacci, Valentin Harter, Pascal Genin, Agnès Leroux, Jean-Louis Merlin

**Affiliations:** 1 Service de Biopathologie, Centre Alexis Vautrin6 Avenue de Bourgogne, 54519, Vandœuvre-lès-Nancy, France; 2 CNRS, UMR 7039 CRANNancy, France; 3 Université de LorraineNancy, France; 4 Service de Biostatistiques, Centre Alexis Vautrin6 Avenue de Bourgogne, 54519, Vandœuvre-lès-Nancy, France

**Keywords:** Colorectal cancer, HRM, *KRAS*, PCR-RFLP, TaqMan PCR

## Abstract

*KRAS* mutation detection represents a crucial issue in metastatic colorectal cancer (mCRC). The optimization of *KRAS* mutation detection delay enabling rational prescription of first-line treatment in mCRC including anti-EGFR-targeted therapy requires robust and rapid molecular biology techniques. Routine analysis of mutations in codons 12 and 13 on 674 paraffin-embedded tissue specimens of mCRC has been performed for *KRAS* mutations detection using three molecular biology techniques, that is, high-resolution melting (HRM), polymerase chain reaction restriction fragment length polymorphism (PCR-RFLP), and allelic discrimination PCR (TaqMan PCR). Discordant cases were assessed with COBAS 4800 *KRAS* CE-IVD assay. Among the 674 tumor specimens, 1.5% (10/674) had excessive DNA degradation and could not be analyzed. *KRAS* mutations were detected in 38.0% (256/674) of the analysable specimens (82.4% in codon 12 and 17.6% in codon 13). Among 613 specimens in whom all three techniques were used, 12 (2.0%) cases of discordance between the three techniques were observed. 83.3% (10/12) of the discordances were due to PCR-RFLP as confirmed by COBAS 4800 retrospective analysis. The three techniques were statistically comparable (*κ* > 0.9; *P* < 0.001). From these results, optimization of the routine procedure consisted of proceeding to systematic *KRAS* detection using HRM and TaqMan and PCR-RFLP in case of discordance and allowed significant decrease in delays. The results showed an excellent correlation between the three techniques. Using HRM and TaqMan warrants high-quality and rapid-routine *KRAS* mutation detection in paraffin-embedded tumor specimens. The new procedure allowed a significant decrease in delays for reporting results, enabling rational prescription of first-line-targeted therapy in mCRC.

## Introduction

Colorectal cancer (CRC) is the second most common cause of cancer with more than one million new cases diagnosed every year [1]. The World Health Organization estimates that 608,000 people die every year from clinical complications and metastasis of CRC.

From 2006 to 2008, several studies showed the importance of the *KRAS* oncogene in the treatment of metastatic colorectal cancer (mCRC) and response to anti-EGFR therapies as cetuximab or panitumumab [2–6]. KRAS is a small G protein, which can bear activating mutations in 40% cases of mCRC [7]. *KRAS* mutations cause RAS protein accumulation in an active state through intrinsic GTPase activity inhibition, which leads to the constitutive activation of the RAS/RAF/MAPK signaling pathway [8]. The most common reported mutations of *KRAS* are on codon 12 (c.35G>A – p.G12D; c.35G>T – p.G12V; c.34G>T – p.G12C; c.34G>A – p.G12S; c.35G>C – p.G12A, and c.34G>C – p.G12R) and codon 13 (c.38G>A – p.G13D) and represent 98.2% of the mutations located in the exon 2 [9]. G13D mutations represent more than 87% of codon 13 mutations according to COSMIC Sanger database. Mutations on codons 61 and 146 have also been described in 2.1% and 1.9% of the cases, respectively [7]. Only one retrospective study showed the impact of codon 61 or codon 146 on response to anti-EGFR therapies in mCRC [10] and only codons 12 and 13 mutations are clearly reported to be predictive of response to cetuximab or panitumumab. *KRAS* mutation detection on codons 12 and 13 is mandatory for the administration of anti-EGFR therapies, as the OPUS [11] and CRYSTAL [12] studies showed that *KRAS* mutations are predictive of response to treatment to cetuximab associated with fluorouracil, leucovorin, and oxaliplatin (FOLFOX) or fluorouracil, leucovorin, and irinotecan (FOLFIRI), respectively. A pooled analysis of both studies showed that addition of cetuximab in wild-type *KRAS* patients with mCRC improves progression-free survival and overall survival [13]. Benefits of panitumumab associated with FOLFOX or FOLFIRI have been described on progression free survival (PFS) in wild-type *KRAS* patients with mCRC [14–16] in first-line treatment, but the OS was only significantly better when associated with FOLFIRI [14]. In second-line treatment, PFS was significantly better when FOLFIRI was associated with panitumumab [17]. Although the EGFR signaling pathway is thought to play a central role in cell proliferation and malignant transformation, no correlation has been shown between EGFR expression and response to treatment. Furthermore, *EGFR* mutations are rare (<1%) in colorectal cancer [18] and had no influence on anti-EGFR response in mCRC and therefore cannot be used to predict the clinical response to anti-EGFR monoclonal antibodies.

The introduction of targeted therapies, that is, anti-VEGF and anti-EGFR monoclonal antibodies, have substantially enriched the therapeutic options in mCRC, and long-term survival (>48 months) can now be achieved in approximately one-third of the patients [19]. In addition, improvement of the rate of resectability of metastases after conversion chemotherapy results in cure for numerous patients [20]. Therefore, the selection of first-line therapy in mCRC is crucial and must be individualized according to the treatment strategy, the patient tumor biology, and the toxicity associated with each therapeutic option. The health authorities regulatory restriction of the prescription of anti-EGFR monoclonal antibodies to wild-type *KRAS* tumor patients plays a major role in selecting anti-VEGF or anti-EGFR introduction in first-line therapy. No present molecular diagnostic has been required or identified for the prescription of the anti-VEGF monoclonal antibody bevacizumab.

Based on this knowledge, routine *KRAS* mutation detection plays a major role in the choice between first-line therapies using anti-EGFR or anti-VEGF monoclonal antibodies: the oncologists need to choose in a rational way the first-line therapy, that is, with all decision-making data being available and not by default because *KRAS* mutation detection results are not available at the time of initiation of the first-line therapy.

There is no standardized method for *KRAS* mutation testing. Sequencing is considered the “gold standard,” but has been reported to suffer from a lack of specificity and sensitivity, justifying extensive evaluation of alternative techniques for routine *KRAS* detection analysis. Recently, the use of sequencing was showed to yield misinterpretation leading to lack of response to anti-EGFR antibodies in mCRC bearing small *KRAS-*mutated DNA content [21,22]. Recent paper by Molinari et al. [23] showed that direct sequencing has a sensitivity of 20%, and this sensitivity can be better using techniques like MALDI-TOF MS, mutant-enriched PCR, or engineered mutant-enriched PCR to 10%, 0.1%, and 0.1%, respectively.

The aim of this study conducted in Alexis Vautrin Cancer Center was to establish a rapid, robust, and sensitive *KRAS* mutations determination testing procedure in order to provide reliable results to the oncologists with shortest delay, contributing to the best care provided to the patients. Some hypotheses regarding the quality on the tumor tissue specimens are also discussed. Thus, we compared high-resolution melting (HRM) analysis, polymerase chain reaction restriction fragment length polymorphism (PCR-RFLP), and TaqMan PCR techniques for determination of *KRAS* mutations, then we compared our data with the literature, and we finally evaluated the impact of the techniques on the delay between analysis prescription by the oncologist and the result reporting.

## Material and Methods

### Study population

A total of 674 paraffin-embedded biopsies and resection specimens from patients with a metastatic colorectal cancer have been collected from academic and private pathology laboratories for routine *KRAS* status assessment in the Alexis Vautrin Cancer Center from January 2008 to December 2009. Of the 674 samples, 582 were from colorectal primitive tumors (86.4%) and 83 from metastatic sites (12.3%). This information was not available for nine samples (1.3%). The sex ratio M/F was 1.51 and the median age was 65.1 years with a range of 24–87.

### DNA extraction

**Figure 1:**
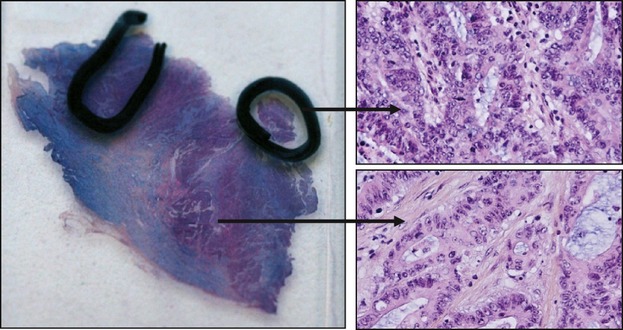
Macrodissection step to ensure a minimum of 20% tumor tissue content. The hematoxylin–eosin slide with selected area contains more than 20% tumor cells.

Tumor specimens were macrodissected after hematoxylin–eosin slide qualification by a pathologist to ensure a minimum of 20% tumor tissue content as recommended by Bibeau et al. (Fig. 1) [24]. Five 10-*μ*m-thick serial sections were cut from each paraffin block and collected in Eppendorf^®^ vials. DNA isolation was performed using the QIAamp DNA FFPE tissue kit (Qiagen, Courtaboeuf, France) protocol. Briefly, paraffin was removed by extraction with toluene and centrifuged. The pellet was then washed with ethanol, centrifuged, and resuspended with 180 *μ*L of tissue lysis buffer (buffer ATL; Qiagen) and 20 *μ*L of proteinase K. The sample was gently mixed, incubated at 56°C for 1 h and 90°C for 1 h under agitation. DNA was extracted with MinElute Columns (Qiagen) according to the manufacturer's recommendations. The nucleic acids were eluted in a volume of 100 *μ*L and diluted to have a final concentration of 20 ng/*μ*L. DNA with identified *KRAS* mutations were used as positive control and known wild-type DNA as negative control.

### TaqMan PCR

The presence of the seven most common *KRAS* mutations in mCRC (codon 12: G12D, G12V, G12C, G12S, G12A, G12R; codon 13: G13D) was determined by TaqMan allelic discrimination assay according to Lievre et al. [4]. Specific probes for each allele (mutated and nonmutated alleles) were labeled, respectively, with the fluorescence reporter dyes FAM and VIC at their 5′-end. Reactions were performed in 5 *μ*L comprising 20 ng of DNA using 384-well plates (Roche Diagnostics, Meylan, France), specific primers and probes, and TaqMan Genotyping Master Mix (Applied Biosystems, Villebon-sur-yvette, France). DNA was then submitted to the following cycle conditions: 95°C for 10 min; 40 cycles, 95°C for 15 sec and 60°C for 1 min. Data were analyzed with LC480 software (Roche Diagnostics). All assays were performed in duplicate.

### High-resolution melting analysis

HRM analysis was performed using the LC 480 HRM Master kit (Roche Diagnostics) and 384-well plates (Roche Diagnostics, Hamburg, Germany) according to Krypuy et al. [25]. HRM analysis allows to detect all the possible somatic mutations of exon 2 including all codons 12 and 13 mutations. Forty nanograms of DNA was amplified in a final volume of 18 *μ*L by using the following: 10 *μ*L of Master Mix HRM LC480, 2 *μ*L of MgCl_2_ (25 mmol/L), 1 *μ*L of primers (4 *μ*mol/L) (Eurogentec, Seraing, Belgium), 2 *μ*L of water. DNA was then submitted to the following cycle conditions: initial denaturation at 95°C for 10 min followed by 45 cycles of 10 sec at 95°C, 15 sec at 67°C, and 10 sec at 72°C. For the HRM melting profile, samples were denatured with an initial hold of 1 min at 95°C and 1 min at 40°C and a melting profile from 65°C to 95°C with a ramping degree of 0.02°C/sec. All assays were performed in duplicate.

### PCR-RFLP

A two-step PCR-RFLP was performed according to Schimanski [26] as previously described [27]. Briefly, 100-ng DNA was used as template for the first PCR (Master Cycler Gradient, Eppendorf, Germany) with the oligonucleotide primers Ras A (sense; 5′-ACTGAATATAAACTTGTGGTCCATGGAGCT-3′) and Ras B (antisense; 5′-TTATCTGTATCAAAGAATGGTCCTGCACCA-3′). PCR products were then submitted to enzymatic digestion with either BstXI or XcmI, restricting the amplicon if the first two bases of codon 12 (BstXI) and codon 13 (XcmI) was wild type. The first digest (2 *μ*L) was used as template for the second PCR in which primer Ras C (antisense; 5′-GGATGGTCCTCCACC AGTAATATGGATATTA-3′) was used instead of Ras B. Second PCR product (7 *μ*L) was digested with either BstXI or XcmI. The digest product (10 *μ*L) was submitted to 4% agarose gel stained with ethidium bromide and analyzed under UV light (GelDoc EQ; Bio-Rad, Hercules, CA). All assesses were processed in a controlled atmosphere room to avoid samples cross-contaminations.

### COBAS 4800 *KRAS*

Discordant cases were retrospectively re-analyzed using the CE-IVD-validated COBAS 4800 *KRAS* TaqMelt assay (Roche Diagnostics). Samples were processed according to the manufacturer's protocol based on previously validated data [28]. Fifty nanograms of previously extracted DNA was dispatched in 96-well plates (Roche Diagnostics), as well as negative and positive controls from the kit. Mutations detection is achieved automatically by the COBAS software achieving melting curves analysis. Amplification detection, quality control analysis, and result interpretation are automated using software package. All samples were processed once, as recommended by the manufacturer.

### Sensitivity

The sensitivity of RFLP, PCR-HRM, and PCR TaqMan assays was evaluated by mixing codon 12 mutated and wild-type DNA from cell lines (A549 as codon 12 mutated, WIDR as wild type) at 100%, 50%, 25%, 10%, 5%, 2.5% and 1% ratios. Same protocol has been followed for the determination of the sensitivity for codon 13 mutations (LOVO as codon 13 mutated, WIDR as wild type).

### Statistics

Significance of the concordance of mutation detection with different methods was assessed by κ statistics. κ superior to 0.8 was considered statistically significant. The chi-square test was used to compare mutation frequencies within data or with those obtained from literature. *P *<**0.05 was considered statistically significant.

## Results

**Figure 2:**
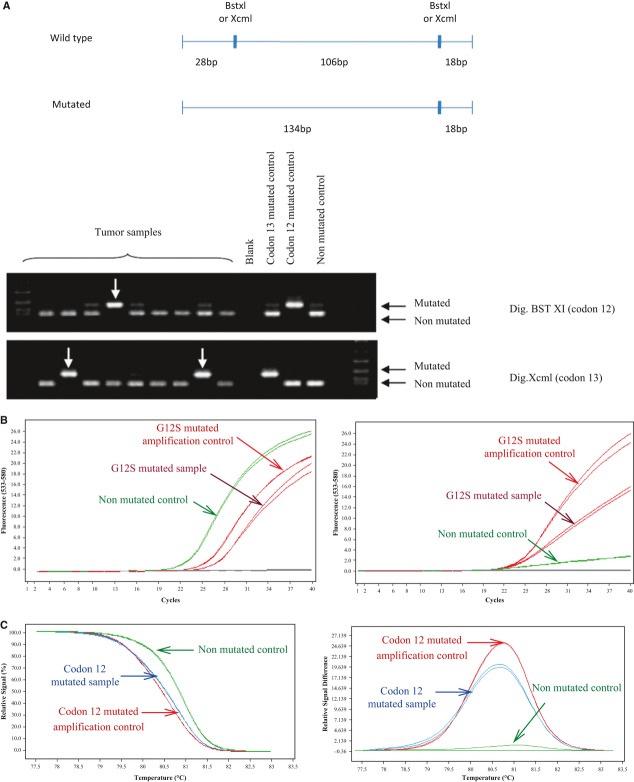
(A) *KRAS* mutation analysis using PCR-RFLP. DNA extracts from tumor samples were submitted to double PCR amplification after BstXI and XcmI enzymatic digestion allowing discrimination of codons 12 and 13 mutations. Codons 12 and 13 mutated DNA were used as positive control. Wild-type *KRAS* DNA and water were used as negative controls. (B) Example of codon 12 *KRAS* mutation detection using TaqMan PCR. Left panel represents amplification control (VIC). Right panel represents G12S mutation detection (FAM). (C) Example of codon 12 *KRAS* mutation detection using HRM. Depending on the presence or the absence of mutation, the melting temperature is different (left panel). The plot of the relative signal difference against the temperature allows to evidence the presence or the absence of *KRAS* mutation (right panel).

*KRAS* somatic mutation detection was assessed for routine diagnostic in a blinded fashion on the 674 samples using TaqMan PCR, HRM PCR, and PCR-RFLP assays (Fig. 2). PCR-RFLP provided 32 (4.7%) of noninterpretable (NI) results, 22 (3.3%) for TaqMan PCR, and 37 (5.5%) for HRM PCR (Table 1). Among the 674 tumor specimens, 1.5% (10/674) remained NI, even combining the results of the three techniques, because of excessive DNA degradation.

**Table 1:** Interpretable, noninterpretable (NI) results and discordances for polymerase chain reaction restriction fragment length polymorphism (PCR-RFLP), TaqMan PCR, and HRM PCR. TaqMan PCR showed less NI results than the two other assays

*n* = 674	PCR-RFLP		TaqMan PCR		HRM PCR	
Interpretable	640	(94.96%)	650	(96.44%)	635	(94.21%)
NI	32	(4.75%)	22	(3.26%)	37	(5.49%)
False positive	4	(0.62%)	0	(0.00%)	0	(0.00%)
False negative	6	(0.93%)	2	(0.31%)	2	(0.31%)

**Figure 3:**
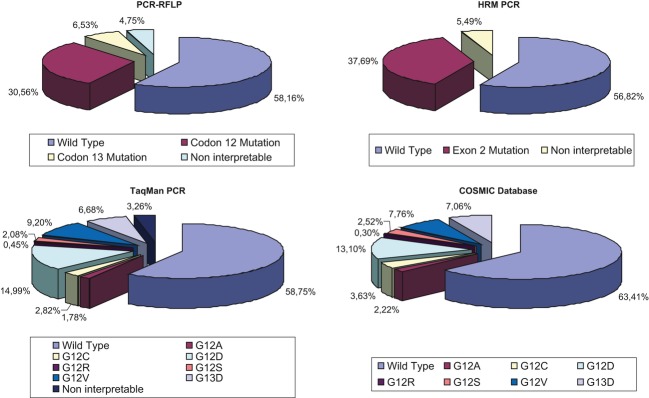
Comparison of mutation frequency as determined by the different detection methods. Overall as well as for each technique, all mutations frequencies were compared with average data from the Sanger Cosmic data base and were found to be fully consistent with the theoretical frequencies (chi-square test nonsignificant for all the data).

Among the 652 interpretable cases, TaqMan PCR revealed 256 cases (39.3%) with mutation in either codon 12 (82.4%) or codon 13 (17.6%) (Fig. 3). Mutations were distributed as follows: 101 G12D mutations (39.4%), 62 G12V mutations G12V (24.2%), 19 G12C mutations (7.4%), 12 G12A mutations (4.7%), 14 G12S mutations (5.5%), 3 G12R mutations (1.2%), and 45 G13D mutations (17.6%). Among the 637 interpretable cases, HRM PCR revealed 254 *KRAS* mutations (39.9%) (Fig. 3). PCR-RFLP revealed 250 of 642 interpretable cases (38.9%) with *KRAS* mutations, among which 206/250 (82.4%) were located in codon 12 and 44/250 (17.6%) in codon 13 (Fig. 3). Overall, as well as for each technique, all mutations frequencies were compared with data extracted from the Sanger Cosmic data base (http://www.sanger.ac.uk/genetics/CGP/cosmic/) and were found to be fully consistent with the reference frequencies (chi-square test nonsignificant for all the data). A comparison of mutations frequencies between resections and biopsies revealed no significant difference, as already evidenced by Weichert et al. (chi-square test data not showed) [29].

A comparison of these three methods yielded κ values exceeding 0.9 (*P *<**0.001), showing an excellent correlation between the three techniques used (Table 2). Among 613 specimens in whom all three techniques were used and gave interpretable results, 12 (2.0%) cases of discordance between the three techniques were observed. In these discordant cases, the complete procedure (from DNA extraction to PCR) was systematically repeated. Discordant samples were processed retrospectively using COBAS 4800 *KRAS* CE-IVD validated method. Mutated samples, detected as nonmutated samples, were considered false negative. Nonmutated samples, detected as mutated samples, were considered false positive. More than 83% (10/12) of discordances were attributable to PCR-RFLP, as revealed by a second analysis of the same sample. Six false-positive samples and four false-negative samples were found. No false positive were found with TaqMan PCR and HRM PCR. Two false negative were found with TaqMan PCR (detected with RFLP, HRM, and COBAS) and two false negative were found with HRM PCR (detected with TaqMan and COBAS) (Table 1).

**Table 2:** Crossover comparison of mutation frequency as determined by polymerase chain reaction restriction fragment length polymorphism (PCR-RFLP), PCR Taqman, and HRM PCR: κ values exceed 0.9 (*P* < 0.001), showing an excellent correlation between the three techniques

	PCR-RFLP	HRM PCR
TaqMan PCR	*n* = 631	*n* = 630
	κ = 0.960	κ = 0.987
	*P* < 0.001	*P* < 0.001
HRM PCR	*n* = 619	
	κ = 0.973	
	*P* < 0.001	

Optimization of the routine procedure consisted of proceeding to systematic *KRAS* detection using HRM and TaqMan PCR instead of simultaneous use of HRM, TaqMan PCR, and PCR-RFLP. The use of PCR-RFLP was restricted to discordant cases. This new operating procedure allowed to significantly reduce the reporting delay on the basis of weekly analyzes from 10.5 ± 7.0 days to 8.5 ± 3.3 days (*P *<**0.001), that is, a 19% decrease.

### DNA quality

Ten samples provided NI results. Among the NI results, 70% of the tumor samples results were provided by 5% of pathology laboratories, probably related to preanalytical procedure (paraffin embedding, fixative, and fixation time) that could be responsive for DNA degradation. Among discordant results, 30% are provided by these 5% laboratories. This may suggest that the preanalytical step could be responsive for increased false-positive or false-negative patients. However, no significant difference was found when comparing the frequency of *KRAS* mutations in different pathology laboratories (data not shown).

### Sensitivity

Samples have been processed with the four assays and compared (Table 3). For RFLP and HRM, limits of sensitivity were 5% of mutated DNA for codon 12. For COBAS, limits of sensitivity were less than 1% of mutated DNA. PCR TaqMan sensitivity was better for G12D, G12V, and G12C (<1% of mutated DNA), G12A (2.5% of mutated DNA), and equal to other assays for G12S and G12R mutations (5% of mutated DNA). Limits of sensitivity for codon 13 were 5% of mutated DNA for RFLP, HRM, 2.5% of mutated DNA for TaqMan PCR, and less than 1% of mutated DNA for COBAS.

**Table 3:** Sensitivity of RFLP, PCR TaqMan, HRM, and COBAS assays. The sensitivity was evaluated by mixing codon 12 or codon 13 mutated and wild-type DNA from cell lines at 100%, 50%, 25%, 10%, 5%, 2.5%, and 1% ratios

	Codon 12						Codon 13
	G12D	G12V	G12C	G12S	G12A	G12R	G13D
RFLP	2.5%						5.0%
TaqMan PCR	<1.0%	<1.0%	<1.0%	5.0%	2.5%	5.0%	2.5%
HRM PCR	5.0%						5.0%
COBAS	<1.0%						<1.0%

## Discussion

Determination of *KRAS* status before prescription of anti-EGFR therapy is mandatory for patients with metastatic colorectal carcinoma (mCRC). The rational selection of the first-line individualized therapy, between anti-EGFR therapy and anti-VEGF therapy, is only possible if the patient benefits of tumor *KRAS* mutation testing, whereas without this determination, the therapeutic choice would be done by default. This improvement of the personalized medicine implicates that the oncologist should be provided with reliable results within delay that is consistent with the clinical management of the patient. Therefore, the optimization of *KRAS* mutations analysis for prescription of cetuximab and panitumumab in mCRC needs rapid and robust molecular biology techniques. However, validated methods and standardized testing procedures are lacking. Here, we report a comparison between three methods for *KRAS* mutation testing: HRM PCR, TaqMan PCR, and PCR-RFLP. These methods are found to be equivalent; however, HRM seems to be accurate enough as already shown by Weichert et al. [29] and represents a fast method for scanning somatic sequence alterations [30]. HRM PCR sensitivity is close to 100% [30–32], but as showed by the two false-negative results found in this study, this assay should be coupled with a more sensitive technique. The identification of the mutations located in codons 12 and 13, for example, 90% of *KRAS* mutations[33] in accordance with European *KRAS* Quality Assurance Program [34], is also a point to focus, according to recent studies suggesting that patients with *KRAS* G13D-mutated tumors could benefits of cetuximab therapy [35,36]. Thus, the use of Taqman PCR allows mutations identification and is reliable and sensitive [4, 37, 38]. In our study, two false negative were detected when compared with other technique which confirm that this technique should be systematically coupled with a nonspecific PCR method as HRM or TaqMelt.

Mutations located in codons 61 and 146 are less frequent and their detection is not mandatory before anti-EGFR antibodies prescription, although prevalence of these mutations is higher than some of the mutations in codon 12 [7, 39, 40]. As functional consequences on RAS protein could be different of codons 12 and 13 mutations, the clinical implication of these mutations remained unclear until recently [6,18]. However, recent studies emphasize the negative impact of these mutations on the response rate to anti-EGFR monoclonal antibodies [10,39] and might highlight the need for simultaneous detection of mutations in codons 12, 13, 61, and 146 using, for example, multiplex amplification of exons 2, 3, and 4, as proposed by several authors [41,42].

In our institute, the new procedure consisting in using HRM and TaqMan PCR in all specimens and to restrict the use of PCR-RFLP to discordant cases was found to significantly reduce the delay of result reporting, but also to improve the accuracy. Combining three mutation testing techniques greatly reduces the probability to get false-negative or false-positive result. The alternative approach consisting in systematically repeating the whole procedure [43] (e.g. confirmation of *KRAS* mutations by two independent analyses) would lead to increases of the reporting delay, consuming of tumor sample, and would probably not overcome the lack of sensitivity or specificity of a single method. The recent Flash-*KRAS* study [44] showed that the mean delay in France for *KRAS* genotyping was 23.6 ± 28.2 days in 2011. This study showed the importance of the choice of an appropriate assay to provide *KRAS* genotyping results in the recommended 2–3 weeks of delay of the French National Cancer Institute (INCa).

This study also emphasized that the preanalytical procedure needs to be strictly controlled as DNA degradation was found to be the main cause of NI results, thus leading to late reporting of the results, as well as an overcost when repeating the analysis. Moreover, these samples are also unusable for other genetic tests. Bouin fixation is a well-known cause of DNA degradation, leading to NI results in almost 100% of the cases. Fixation duration has been shown to be a critical parameter as well: Inoue et al. [45] demonstrated that if the samples are fixed in 10% nonbuffered formalin for 1 day, 100% of samples show successful PCR, while only 44% of samples show successful PCR when fixed for 2–3 days. More recent studies also showed the influence of the fixative choice, the paraffin temperature, and warm and cold ischemia on the DNA quality for molecular biology [46,47]. In addition, several recent studies reported that paraffin embedding and fixation procedures may induce nucleotide changes through deamination of cytosine and adenine. These deaminations generate uracil and hypoxanthine, respectively, and lead to artifactual C>T and G>A transitions and provide false-positive results [48,49]. This can partly explain discordances observed in our study. Indeed, Marchetti et al. described [50] repeated 10 PCR amplifications on clinical samples. For a same sample, the presence or the absence of uracil could lead to artifactual mutations only in some of the PCR products, thus leading to a false-positive result. Beside this phenomenon, discordances can also be explained by the difference of sensitivity and specificity of the different methods.

Therefore, the preanalytical steps should be optimized and controlled in order to warrant the quality of nucleic acids.

Beside fixation, the step of macrodissection has been reported to be highly critical [48]. According to the authors, the detection rates of mutations clearly decreased with the percentage of tumor cells, and the limit of 20% of tumor material seems to be critical, with a dramatic increase in the risk of false-negative results. In our study, all samples were macrodissected in order to ensure a minimum of 20% of tumor material, and the percentages of each mutations were found to be fully consistent with literature data [51].

Following the question of samples with low cellularity (particularly in the case of neoadjuvant treatment), some authors recommend that a biopsy should be always dedicated to molecular biology before treatment, or to replace the step of macrodissection by laser microdissection [48].

In case of NI results with all assays, a new sample originating from a different tumor site is requested if available.

To conclude, this study emphasizes the fact that *KRAS* mutations analysis in mCRC needs quality control procedures from preanalytical to analytical steps. Our experience using HRM and TaqMan PCR in routine shows that reporting delays suitable with the oncologist expectation could be achieved and enable rational, fully documented, selection of first-line therapy in mCRC. However, with only approximately 50% of the patients responding to anti-EGFR monoclonal antibodies, it should be kept in mind that *KRAS* status has a poor positive predictive value [52]. This emphasizes the need for additional response predictive markers to improve the selection of potential responders among wild-type patients [24,53]. Recent studies showed that *KRAS* mutation detection with more sensitive method allow a better selection of patients who could benefit of anti-EGFR therapies [21,22]. Data reported by Molinari et al. [23] emphasize the importance of sensitivity of the technique: in this paper, authors found 55% of wild-type *KRAS* with standard assay. After using more sensitive assays like MALDI-TOF MS, mutant-enriched PCR, or engineered mutant-enriched PCR, the authors found 27% of these samples identified as wild type bearing a *KRAS* mutation. Our data are consistent with the results of this paper and confirm the importance of a rapid and sensitive assay to avoid false negative. To our opinion, the real-time PCR assays allow to achieve relatively high sensitivity using simple and affordable techniques easily accessible for routine analysis.
